# The Correlation of Dyslipidemia with the Extent of Coronary Artery Disease in the Multiethnic Study of Atherosclerosis

**DOI:** 10.1155/2018/5607349

**Published:** 2018-03-27

**Authors:** Moshrik Abd alamir, Michael Goyfman, Adib Chaus, Firas Dabbous, Leslie Tamura, Veit Sandfort, Alan Brown, Mathew Budoff

**Affiliations:** ^1^Advocate Lutheran General Hospital, Parkside Ste B-01, 1775 Dempster St, Park Ridge, IL 60068, USA; ^2^Stony Brook University Hospital, 101 Nicolls Rd, Stony Brook, NY 11794, USA; ^3^James R. & Helen D. Russell Institute for Research & Innovation, Advocate Lutheran General Hospital, Center for Advanced Care, 1700 Luther Lane, Suite 1410, Park Ridge, IL 60068, USA; ^4^National Institute of Health, 30 Convent Dr, Bethesda, MD 20892, USA; ^5^Harbor UCLA Cardiology, 1000 W. Carson St, Torrance, CA 90509, USA

## Abstract

**Background:**

The extent of coronary artery calcium (CAC) improves cardiovascular disease (CVD) risk prediction. The association between common dyslipidemias (combined hyperlipidemia, simple hypercholesterolemia, metabolic Syndrome (MetS), isolated low high-density lipoprotein cholesterol, and isolated hypertriglyceridemia) compared with normolipidemia and the risk of multivessel CAC is underinvestigated.

**Objectives:**

To determine whether there is an association between common dyslipidemias compared with normolipidemia, and the extent of coronary artery involvement among MESA participants who were free of clinical cardiovascular disease at baseline.

**Methods:**

In a cross-sectional analysis, 4,917 MESA participants were classified into six groups defined by specific LDL-c, HDL-c, or triglyceride cutoff points. Multivessel CAC was defined as involvement of at least 2 coronary arteries. Multivariate Poisson regression analysis evaluated the association of each group with multivessel CAC after adjusting for CVD risk factors.

**Results:**

Unadjusted analysis showed that all groups except hypertriglyceridemia had statistically significant prevalence ratios of having multivessel CAC as compared to the normolipidemia group. The same groups maintained statistical significance prevalence ratios with multivariate analysis adjusting for other risk factors including Agatston CAC score [combined hyperlipidemia 1.41 (1.06–1.87), hypercholesterolemia 1.55 (1.26–1.92), MetS 1.28 (1.09–1.51), and low HDL-c 1.20 (1.02–1.40)].

**Conclusion:**

Combined hyperlipidemia, simple hypercholesterolemia, MetS, and low HDL-c were associated with multivessel coronary artery disease independent of CVD risk factors and CAC score. These findings may lay the groundwork for further analysis of the underlying mechanisms in the observed relationship, as well as for the development of clinical strategies for primary prevention.

## 1. Introduction

The total CAC score (Agatston score) using noncontrast computed tomography is a recognized estimation of atherosclerosis in asymptomatic adults with at least moderate risk of cardiovascular disease [1–3]. When compared with a risk-stratification tool such as the Framingham Risk Score (FRS), the CAC score has been shown to have a superior role in predicting future cardiac events and all-cause mortality [2, 4]. Previous studies demonstrated that measurement of coronary artery calcium stratified patient risk for cardiovascular disease regardless of dyslipidemia burden or definition [5]. However, the relationship between subclinical atherosclerosis and dyslipidemia type has been investigated using either the prevalent CAC or carotid thickness as surrogate markers [5, 6]. Although the traditional Agatston CAC score is a powerful predictor of mortality, there is an emerging evidence that extent of subclinical atherosclerosis, as indicated by the number of vessels with CAC, further improves cardiovascular risk prediction [2, 7]. With the current ability to identify those with multivessel disease at an asymptomatic stage using the CAC, the healthcare providers could potentially tailor the diagnostic and therapeutic approach for individual patients based on the extent of CAC. Therefore, the purpose of this study was to determine whether there is an association between common dyslipidemias (combined hyperlipidemia, simple hypercholesterolemia, MetS, isolated low high-density lipoprotein cholesterol and isolated hypertriglyceridemia) compared with normolipidemia, and the extent of coronary artery involvement (multivessel CAC), among MESA participants who were free of clinical cardiovascular disease at baseline. It remains to be seen whether the association between dyslipidemia types and the CAD extent still exists after controlling for the absolute calcium score.

## 2. Methods

### 2.1. Study Cohort

The Multiethnic Study of Atherosclerosis (MESA) is a prospective observational evaluation of 6,814 men and women, aged 45 to 84 years, from four racial/ethnic groups (White, Asian, Hispanic, and Black) in the United States. At the time of enrollment, participants had no known cardiovascular disease.

Participants were enrolled between July 2000 and September 2002 at field centers located in Forsyth County, North Carolina; St. Paul, Minnesota; Chicago; New York City; Baltimore, Maryland; and Los Angeles. Institutional review boards approved the study protocol at each study center. Further details regarding the MESA study have been detailed elsewhere [8].

Study participants provided information about cardiovascular risk factors. A central laboratory (University of Vermont, Burlington, Vermont) measured levels of total and high-density lipoprotein cholesterol, triglycerides, plasma glucose, and high-sensitivity C-reactive protein (CRP) after a 12-hour fast. The coronary calcium scan was done twice in each participant to increase accuracy. For each scan, the participant was asked to remain still and momentarily hold his/her breath twice, each time for 20 to 30 seconds, in order to get good quality pictures. Participants' CAC scores were reported as average CAC Agatston scores. Vessel-specific CAC scores were calculated in 6,540 MESA participants (96%). Of those, 6,479 MESA participants with coronary calcium CT scans at baseline and vessel-specific CAC distribution (left main, left anterior descending, left circumflex, and right) were screened. Included in the final study cohort were 4,917 participants, after exclusion of 1562 participants who were taking lipid-lowering medication as well as diabetics and those who lacked measurements of dyslipidemia and/or CAC. Individuals with triglycerides > 500 were not specifically excluded from the analysis. However, in those cases it is usually not possible to calculate LDL-c if using the Friedewald formula, and patients without LDL-c values would have been excluded from our analysis.

Cardiovascular risk factors were measured or collected, and included height, weight, and waist circumference, medical history including presence of diabetes (using the 2003 American Diabetes Association criteria), hypertension (defined as systolic blood pressure > 139 mm Hg at baseline visit, or diastolic blood pressure > 89 mm Hg, or by a history of physician diagnosed hypertension and taking a medication for hypertension), and assessment of personal habits such as alcohol and tobacco use [9–11].

### 2.2. Exposure Variables

The following cardiovascular risk factors were collected at MESA field centers: height, weight, waist circumference, alcohol and tobacco use, family history of heart attack, CAC score, diabetes, and hypertension (systolic blood pressure ≥ 140 mmHg at baseline visit, diastolic blood pressure ≥ 90 mmHg, or a history of taking an antihypertensive medication). Age and race/ethnicity were self-reported. Lipids, including total and high‐density lipoprotein cholesterol, triglycerides, inflammatory markers, and glucose levels, were measured from fasting plasma samples in a central laboratory (University of Vermont, Burlington, VT). Venous blood samples were collected after a 12 h fast by certified technicians using standardized venipuncture procedures. Samples were then centrifuged at 2000*g* for 15 min at 4°C within 30 min of collection. EDTA plasma samples were aliquoted on ice, stored at −70°C, and then shipped on dry ice to the MESA central laboratory at University of Vermont [12].

### 2.3. Dyslipidemia

Table 1 identifies the various HDL-c, LDL-c, and triglyceride categories defining the six mutually exclusive dyslipidemias, including normolipidemia as a reference group.

We created these dyslipidemia groups using criteria based on current National Cholesterol Education Program (NCEP)/Adult Treatment Panel- (ATP-) III guidelines that define LDL-C, HDL-C, and triglyceride thresholds as abnormal [13].

Participants were classified based on the most severe dyslipidemia. For example, someone would be classified in the only MetS if the person had low HDL-c and elevated triglycerides; the person would not be in the low HDL-c group. To define the subtype of dyslipidemia appropriately, we had to exclude diabetes and lipid-lowering therapy from this analysis. Diabetes is considered a coronary heart disease risk equivalent and there is a strong independent relationship of diabetes with low HDL-c, elevated triglycerides, and CAC extent. Participants receiving lipid-lowering therapy were excluded because lipid lowering has a substantial impact on all lipid parameters as well as on CAC. Fasting triglycerides were measured in plasma using a glycerol blanked enzymatic method developed by Trig/GB (Roche Diagnostics, Indianapolis, Indiana). Cholesterol and HDL-c were measured in plasma on the Hitachi 911 using a cholesterol esterase, cholesterol oxidase reaction (Chol R1, Roche Diagnostics). For triglyceride levels < 400 mg/dL, the LDL-c was calculated using the Friedewald formula; otherwise nuclear magnetic resonance spectroscopy was used for triglycerides > 400 mg/dl [9, 14]. Serum glucose was measured by rate reflectance spectrophotometry using thin film adaptation of the glucose oxidase method on the Vitros analyzer (Johnson & Johnson Clinical Diagnostics Inc., Rochester, NY). The laboratory analytical CV was 1.1%. Insulin was determined by a radioimmunoassay method using the Linco Human Insulin Specific RIA Kit (Linco Research Inc., St. Charles, MO). The laboratory analytical CV was 4.9%. CRP was measured in the Laboratory for Clinical Biochemistry Research at University of Vermont (Burlington, VT). CRP was determined using the BNII nephelometer (N High-Sensitivity CRP and N Antiserum to Human Fibrinogen; Dade Behring Inc., Deerfield, IL) [12].

### 2.4. Coronary Artery Calcium (CAC) Extent

Electron-beam computed tomography (EBT) or multidetector row helical computed tomography (MDCT) was used to measure CAC, defined by a minimum of 130 Hounsfield units [10]. The effective dose of radiation for CAC scoring was approximately 1 mSv [11]. The protocol and interpretation of CAC scans in the MESA study have been reported previously [10]. Interobserver and intraobserver agreement were high [15]. The Agatston scoring method quantified baseline CAC [16], and scores were adjusted with a standard biweekly phantom scan to ensure equivalence among sites [15].

Extent of CAC was analyzed according to the number of main coronary arteries (left main, left anterior descending, left circumflex, and right) with calcification ranging from 0 to 4. Multivessel CAC was defined as involvement of at least 2 coronary arteries. This included three-vessel CAC, which was defined as involvement of the left main or left anterior descending coronary artery in addition to CAC in the left circumflex and the right coronary arteries. Single vessel CAC was classified as a distinct entity apart from no CAC and from multivessel disease. Our statistical analysis focused on the relationship between dyslipidemias and specifically multivessel disease, as relationships between dyslipidemias and CAC score in general have previously been described [6].

### 2.5. Statistical Analysis

A cross-sectional sample of participants from the MESA cohort was classified into 6 mutually exclusive dyslipidemia categories (including “normal” as reference group) based on their levels of LDL-c, HDL-c, and triglycerides (Table 1). Differences in baseline demographic and cardiac risk factor data (age, race/ethnicity, gender, clinical site, education, history of hypertension, current smoking status, alcohol use, waist circumference, fasting glucose, fasting insulin, CRP, and creatinine) were evaluated across the 6 lipid categories, using the Chi-Square test for categorical variables and ANOVA for continuous numerical variables. The latter were reported as mean/standard deviation, with the exception of skewed variables including CAC score, serum insulin, CRP, and triglyceride levels, which were reported as median/interquartile range. Multivariate Poisson regression analysis was performed to assess the relationship between multivessel CAC and the type of dyslipidemia (including normal). This was performed both unadjusting and adjusting for the aforementioned demographic and cardiac risk factor data, including the total phantom-adjusted coronary artery calcium (CAC) score. We chose widely accepted parameters associated with cardiovascular risk that were also available in the MESA database. Final model was adjusted for age, gender, race, high-school education, smoking, hypertension, waist circumference, serum glucose level, serum insulin, serum CRP level, and Agatston's calcium score. All variables were adjusted simultaneously. The model based on the chosen variables reflects the well-recognized risk factors associated with CAC score. All the current variables in our final model are literature-derived risk factors. We performed the forward and backward selection to determine the composition of variables for a good model fit and there was no difference with either method. In addition, the *p* value for interaction between different variables such as gender, race/ethnicity, and CRP and lipid variables was not significant for common or internal CIMT or prevalent CAC in the previously published MESA analysis by Paramsothy et al. [6]. A two-sided *p* value < 0.05 was considered to be significant. Data were analyzed using SAS version 9.3 (SAS Institute, Cary, NC).

## 3. Results

### 3.1. Baseline Characteristics

The baseline characteristics of the study cohort are shown in Table 2.

The majority of the cohort consisted of white (39%) and female (53%) participants, with an average age of 61.6 years. Most participants completed at least a high-school level of education (83%) and currently used alcohol (58%). Only 14% of participants were current smokers. Approximately 39% of the cohort had a history of hypertension, and 53% of the cohort had one of the five types of dyslipidemia.

### 3.2. Dyslipidemia

The low HDL-c dyslipidemia group was the most common type of dyslipidemia, followed by the MetS group. The latter had the largest waist circumference (Table 2).

### 3.3. CAC Findings

**Figure 1:**
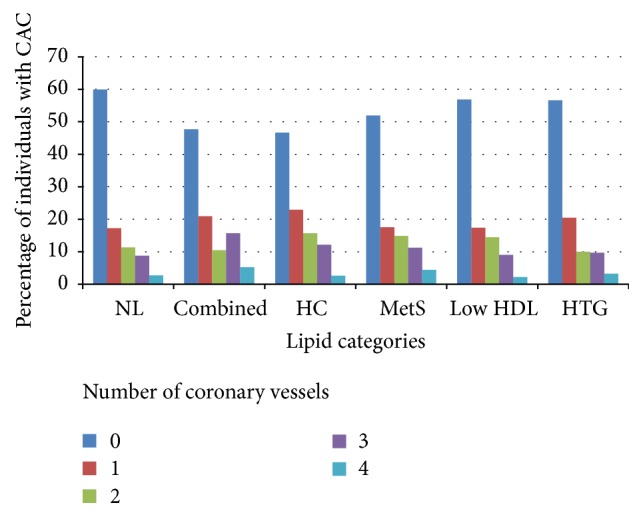
Number of coronary vessels with calcification as a function of lipid profiles. The between-group differences in the number of affected vessels with CAC, showing significant between-group differences (*p* < 0.001). NL = normolipidemia, Combined = combined hyperlipidemia, HC = hypercholesterolemia, MetS = dyslipidemia compatible with metabolic syndrome, HTG = hypertriglyceridemia, and CAC = coronary artery calcification.

A comparison was performed using Chi-Square test between the lipid groups and the number of affected vessels with CAC, showing significant between-group differences (*p* < 0.001, Figure 1).

The normolipidemia group had the highest percentage of individuals with zero affected vessels (60%), whereas the combined hyperlipidemia group had the highest percentage of those with three- and four-vessel calcification (16% and 5%, resp.).

Unadjusted univariate Poisson regression analysis showed that combined hyperlipidemia, simple hypercholesterolemia, and dyslipidemia of metabolic syndrome had a statistically significant likelihood of having a multivessel CAC as compared to the normolipidemia reference group (Table 3).

By contrast, there was no statistically significant difference between the low HDL-c, hypertriglyceridemia, and normolipidemia groups in the prevalence of multivessel CAC in the unadjusted model. Subsequent multivariate Poisson regression analysis adjusting for the demographic and cardiac risk factors, including Agatston calcium score, showed that the same lipid groups (combined hyperlipidemia, simple hypercholesterolemia, and dyslipidemia of metabolic syndrome) and the multivessel CAC maintained statistical significance in the model, compared to the normolipidemia reference group (Table 4). Interestingly, the previously nonsignificant HDL have become significant in the adjusted model. Furthermore, higher Agatston calcium score, age, male gender, Asian race, currently smoking, hypertension, and waist circumference were all significantly associated with multivessel disease. The other demographic and cardiac risk factors previously mentioned were not found to have a significant relationship with multivessel disease.

## 4. Discussion

Previous literature has shown that different types of dyslipidemia have varying association with CAC scores [2, 3, 6, 17]; however, our study elucidates a relationship between the different dyslipidemia types and the extent of coronary artery disease, defined as the rate of multivessel CAC. We focus on whether or not the heterogeneities of baseline subclinical atherosclerotic extent have any relationship with dyslipidemia type and, if so, which dyslipidemia is associated with increased rates of multivessel CAC. As expected, the majority of the normolipidemia group had no CAC-affected vessels. In comparison, all dyslipidemia groups except for the hypertriglyceridemia were associated with higher rates of multivessel CAC. With further adjustment for demographic and cardiac risk factors, the same dyslipidemia groups were still associated with multivessel CAC, even after controlling for the total CAC score. An earlier study by Paramsothy et al. showed that, of 4,795 MESA participants, without known clinical cardiovascular disease, those with combined hyperlipidemia, hypercholesterolemia, and MetS had increased relative risk for prevalent CAC [6] compared with normolipidemia participants when adjusting for demographic and CVD risk factors.

Similar to Paramsothy et al., we found that isolated hypertriglyceridemia was not associated with CAC extent [6]. These findings suggest that an isolated elevation in triglyceride levels may not be a pathologic risk factor for subclinical atherosclerosis, although hypertriglyceridemia may still be an important factor in cardiovascular disease. The hypertriglyceridemia group in this study also had a relatively high HDL-c of 53.6 mg/DL, which may have inversely affected overall CAC prevalence [6].

In contrast to the Paramsothy study where no significant association was found between low HDL-c and prevalent CAC, we found an association between low HDL-c and increased extent of CAC. The prevalence of low HDL-c group and adjusted risk factors were identical in both studies. In their study of 6093 participants, Allison and Wright showed that the individuals with an HDL-c level < 40 mg/dl had significantly higher calcium scores while increases in HDL-c were associated with a significant reduction in risk for the presence of any calcified plaque. Multivariate logistic regression revealed that HDL-c is predictive of calcified plaque development independent of LDL-c. However, sensitivity and positive predictive values for HDL-c were low [18]. Furthermore, Noda et al. studied two hundred and eighty-nine consecutive patients who underwent 64-slice multidetector CT for suspected coronary artery disease. They found that HDL-c cholesterol levels were more accurate for diagnosing the presence of high-risk coronary plaque with areas under receiver operating curve (AUC) of 0.840 in patients with CCS 0, than with AUC of 0.633 in those with CCS 1 to 10, 0.605 in those with CCS 11 to 100, 0.591 in those with CCS 101 to 400, and 0.571 in those with CCS > 400 [19]. Therefore, in participants with lower CAC score, low HDL-c may have more influence on the extent of CAC than those with higher calcium score. This may explain the significance of HDL-c levels found in our study, since, unlike Paramsothy et al., we controlled for CAC score and focused on the extent of CAC.

Combined hyperlipidemia, simple hypercholesterolemia, and participants with MetS had significantly increased risk of multivessel involvement of the coronary arteries in patients with subclinical CAD [20]. This is in line with Paramsothy et al.'s findings where the same dyslipidemia groups were associated with prevalent CAC. Given these findings, elevated LDL-c seems to be the principal determinant of CAC prevalence and its extent, with high TG levels having less influence [6, 17, 20, 21]. Of notice, in the multivariate analysis, the confidence interval of the combined dyslipidemia group is rather wide because the numbers of participants with this disorder are relatively small, 172 (3.5%), influencing the precision of the population estimate.

Atherogenic dyslipidemia is largely underdiagnosed and undertreated in clinical practice per the findings of Fruchart et al. in the Residual Risk Reduction Initiative (R3i). These are prevalent in patients with type 2 diabetes, metabolic syndrome, and/or established CVD. Especially in MetS, these patients commonly had elevated ApoB levels, smaller LDL particle size, and elevated ApoCIII levels and as such metabolic syndrome is associated with residual CVD risk [22].

Gender may also contribute to CAC extent, as we found CAC extent was significantly increased among men. Male gender is a recognized independent risk factor for coronary heart disease by the Framingham Risk Score. In addition, in their study of 6814 participants, McClelland et al. found that men had greater calcium levels than women [15]. The same study showed that calcium amount and prevalence were steadily higher with increasing age and in Whites, whereas Asians had the highest ratio of multivessel CAC in our study.

Coronary artery calcium, a known marker of coronary atherosclerotic plaque, has been consistently associated with cardiovascular morbidity and mortality [2, 15, 17, 23, 24]. The clinical significance of the demonstrated association between dyslipidemia types and the extent of CAC, controlling for CAC score, remains unclear.

Although the absence of CAC is not reassuring in symptomatic patients, the CAC score may be associated with myocardial perfusion defects in asymptomatic patients. Studies have shown that CAC burden and extent predict future coronary revascularization procedures [24]. As Budoff et al. first demonstrated, both the number of calcified vessels and general CAC prevalence are independently associated with increased likelihood of significant angiographic disease [25]. Moreover, CAC prevalence, adjusting for CAC score, was predictive of the mode of revascularization. Independent predictors of coronary artery bypass graft versus percutaneous coronary intervention included three- or four-vessel CAC, higher CAC burden, and involvement of the left main coronary artery.

The significant association between dyslipidemia types, except hypertriglyceridemia, and the extent of CAC may prompt more aggressive treatment and prevention of elevated LDL-c, low HDL-c, combined hyperlipidemia, and MetS. Simple hypercholesterolemia may have the greatest impact in determining the severity of atherosclerotic disease, especially in those with diabetes and taking lipid-lowering medications. As others have shown, LDL-c is the dominant lipid determinant of atherosclerotic disease [6, 17].

As the MESA study gathered data from a large, multiethnic population, the results may be widely generalizable. The imaging and laboratory procedures were standardized at a common institution. Our study also has several limitations. Although we attempted to adjust for all possible confounding factors in our model, the residual confounding by unevaluated factors cannot be completely ruled out. As we examined cross-sectional associations, the possibility of temporal and selection biases may exist. Participants on statin therapy and with diabetes were excluded because these factors could potentially misclassify the lipid categories and confound the relationship between dyslipidemia and CAC. We used the term dyslipidemia compatible with metabolic syndrome instead of dyslipidemia of metabolic syndrome. To define this category, we did not factor in obesity or blood pressure to isolate the impact of dyslipidemia on the extent of subclinical atherosclerosis.

The results of this study further elucidate the role of CAC in cardiovascular disease. Clinicians already use the CAC score to predict future cardiovascular morbidity and mortality in asymptomatic patients with moderate risk factors. Callister et al. [26] showed that CAC scoring might help assess the effects of statin therapy. Budoff et al. illustrated a relationship between coronary calcium extent and CABG [25]. Recognizing the extent of coronary artery calcium may provide additional information beyond whether or not atherosclerotic disease is present. The association of dyslipidemias with CAC extent is further evidence in support of the importance of dyslipidemia, especially simple hypercholesterolemia, as a target for therapy.

The association between multivessel coronary artery disease and the cardiovascular risk factors, including dyslipidemia, had been widely studied in patients with documented clinical CVD, using invasive coronary angiography [27, 28]. Our study is novel to address this relationship, assessed by cardiac computed tomography, in a large multiethnic cohort free of clinical CVDs at baseline. We believe the underlying mechanisms of these associations should be relevant to disease prevention and require further investigation. Furthermore, the current findings may explain the difference when comparing the results of different studies. For instance, in patients with low HDL-c, although the CVD risk is high independently of other cardiovascular risk factors, the clinical trials have shown lack of improvement in the cardiovascular outcome when using drug therapies to boost the level of HDL-c. Our observation highlights the fact that the relative impact of low HDL-c, and potentially the targeting pharmacotherapy, on CAD extent may vary depending on the population selected, low versus high calcium score. Similarly, CVD outcome studies with triglyceride-lowering agents have produced inconsistent results, meaning that no convincing evidence is available that lowering triglycerides by any approach can reduce mortality. The association between plasma TG levels (both fasting and nonfasting) and CV risk is often attenuated once adjusting for other lipid parameters. However, it is worthy to notice the lack of association between high TG level and CAC extent existing in our study even prior to adjusting for other lipid parameters. A number of studies have found that the association between plasma TG levels (both fasting and nonfasting) and CV risk is often attenuated once adjusting for other lipid parameters, including HDL-c and non-HDL-c. An analysis conducted by the Emerging Risk Factors Collaboration demonstrated that there was a significant and stepwise association between fasting and nonfasting TG levels and CVD risk. However, this association was no longer significant after adjustment for HDL-c and non-HDL-c [29]. Elevated TG levels are closely associated with higher levels of non-HDL-c and apoB and low levels of HDL-c, and this may explain why this association is weakened after adjustment for these parameters. This is inherent in the study design since we classified the study cohort into 6 mutually exclusive dyslipidemia categories.

Further research is needed in examining coronary artery calcium extent, not simply the score, as a potential tool in prognosticating, treating, and perhaps preventing subclinical atherosclerosis in otherwise low-risk populations. Other lipid parameters such as non-HDL-c and lipoprotein(a) (Lp(a)) have been proposed as independent risk factors for coronary heart disease and their impact on the extent of multivessel CAC needs to be explored further. A previous study using the MESA population showed that non-HDL-c is independently associated with increased CAC in patient populations without CAC at baseline and especially non-HDL-c > 190 is associated with a significant progression of CAC [30]. Nonetheless, we have identified the 5 major dyslipidemia groups in our analysis based on current National Cholesterol Education Program (NCEP)/Adult Treatment Panel- (ATP-) III guidelines that define LDL-C, HDL-C, and triglyceride thresholds as abnormal. These groups account for the complexity of having more than 1 abnormal lipid parameter in the individual patients. It is challenging to create another mutually exclusive group by using non-HDL-c cutoff because this parameter encompasses all of the circulating atherogenic lipoproteins, including LDL-c. Furthermore, the aforementioned study included participants with diabetes and lipid-lowering medications, whom we excluded in the current analysis, to avoid the effect of these 2 factors on lipid parameters and CAC.

Genetic studies and multiple epidemiologic studies have identified Lp(a) as a risk factor for atherosclerotic diseases such as coronary heart disease and stroke [31]. In a previous study of 410 outpatients, Jug et al. showed that LP(a) is an independent predictor of coronary calcium (odds ratio 7.81, 95% confidence interval 1.41 to 43.5) [32]. In another study by Cho et al., participants with Lp(a) level ≥ 50 mg/dL had an odds ratio of 1.333 (95% CI 1.027–1.730) for CAC progression compared to those with Lp(a) < 50 mg/dL after adjusting for confounding factors [33]. Future investigations testing the association between the Lp(a) and CAC extent in population free of CVD at baseline are warranted. In addition, a published study of MESA showed that the measures of CAC burden and distribution each are independently predicting need for percutaneous coronary intervention versus CABG over an 8.5-year follow-up [2]. The new ACC/AHA 2013 guidelines support intensifying statin therapy when the CAC score is ≥75th percentile for age, gender, and ethnicity/race or when the calcium score is ≥300 Agatston units [34]. Whether adding the extent of CAC, single versus multivessel CAC, to the absolute calcium score poses any incremental therapeutic value needs to be tested in a large population-based cohort.

## 5. Conclusion

In a population-based cohort, the extent of multivessel CAC was associated with different dyslipidemia types except for hypertriglyceridemia. The results were still significant even after controlling for CAC score. Future research should focus on the mechanistic understanding of this relationship for disease prevention and investigate the association between other promising lipid parameters and CAC extent in asymptomatic adults with subclinical atherosclerosis.

## Figures and Tables

**Table 1:** Lipid categories defined by HDL, LDL, and triglyceride levels.

Dyslipidemia category	HDL (mg/dL)	LDL-c (mg/dL)	Triglycerides (mg/dL)
Normolipidemia	>40 men	<160	<150
	>50 women		
Combined hyperlipidemia	No cutoff	≥160	≥150
Hypercholesterolemia	No cutoff	≥160	<150
Dyslipidemia compatible with metabolic syndrome (MetS)	≤40 men	<160	≥150
	≤50 women		
Low HDL-c	≤40 men	<160	<150
	≤50 women		
Hypertriglyceridemia	>40 men	<160	≥150
	>50 women		

**Table 2:** Baseline characteristics and cardiac risk factors of MESA cohort excluding those on lipid-lowering medications and diabetics (collected 2000–2002).

Variable	All	Normal	Combined	HC	MetS	Low HDL	HTG	*p* value
*N (%)*	4917	2329 (47.4)	172 (3.5)	345 (7.0)	793 (16.1)	907 (18.5)	371 (7.6)	
*Age (SD)* ^*∗*^	61.6 (10.3)	62.3 (10.5)	61.9 (9.9)	61.4 (9.5)	60.8 (10.3)	60.8 (10.6)	61.5 (9.4)	0.015
*Male gender (%)* ^*∗*^	2311 (47.0)	1054 (45.3)	74 (43.0)	155 (44.9)	438 (55.2)	406 (44.8)	184 (49.6)	<0.001
*Race/ethnicity* ^*∗*^								<0.001
White (%)	1930 (39.3)	960 (41.2)	70 (40.7)	135 (39.1)	311 (39.2)	282 (31.1)	172 (46.4)	
Asian (%)	616 (12.5)	279 (12.0)	20 (11.6)	31 (9.0)	126 (15.9)	117 (12.9)	43 (11.6)	
Black (%)	1285 (26.1)	700 (30.1)	24 (14.0)	113 (32.8)	96 (12.1)	303 (33.4)	49 (13.2)	
Hispanic (%)	1086 (22.1)	390 (16.8)	58 (33.7)	66 (19.1)	260 (32.8)	205 (22.6)	107 (28.8)	
*Completed high school (%)* ^**∗**^	4079 (83.0)	2029 (87.1)	136 (79.1)	285 (82.6)	609 (76.8)	730 (80.5)	290 (78.2)	<0.001
*Current smoker (%)* ^**∗**^	690 (14.0)	296 (12.7)	21 (12.2)	46 (13.3)	143 (18.0)	129 (14.2)	55 (14.8)	0.011
*Hypertension (%)* ^**∗**^	1903 (38.7)	874 (37.5)	62 (36.1)	127 (36.8)	339 (42.8)	336 (37.1)	165 (44.5)	0.014
*Waist circumference, cm (SD)* ^**∗**^	96.8 (14.0)	93.6 (14.4)	100.2 (12.4)	97.0 (12.7)	101.4 (12.6)	99.3 (13.5)	99.0 (13.1)	<0.001
*Fasting glucose, mg/dL (SD)* ^**∗**^	91.1 (19.2)	88.5 (13.6)	95.8 (26.0)	92.0 (21.1)	96.4 (27.9)	91.8 (18.9)	92.0 (19.3)	<0.001
*Insulin, mU/L* ^**∗**^	7.9 (11.4–5.8)	6.7 (9.1–5.0)	9.1 (13.6–6.9)	7.7 (11.0–5.8)	10.9 (14.9–8.0)	9.1 (12.6–6.5)	8.6 (12.5–6.5)	<0.001
*CRP, mg/L* ^**∗**^	1.9 (4.2–0.8)	1.5 (3.7–0.7)	2.4 (5.7–1.2)	1.9 (4.2–1.0)	2.3 (4.4–1.1)	2.2 (4.8–0.9)	2.4 (5.8–1.1)	0.0001
*Creatinine, mg/dL (SD)*	0.95 (0.22)	0.94 (0.20)	0.94 (0.19)	0.96 (0.19)	0.96 (0.24)	0.94 (0.26)	0.94 (0.23)	0.352
*Total cholesterol, mg/dL (SD)* ^**∗**^	197.1 (35.3)	190.4 (27.1)	262.9 (23.2)	250.0 (22.2)	197.1 (33.4)	175.7 (26.5)	212.0 (30.6)	<0.0001
*LDL, mg/dL (SD)* ^**∗**^	120.4 (31.0)	112.7 (24.4)	177.2 (18.4)	177.2 (18.8)	113.8 (25.7)	114.6 (24.0)	116.3 (25.1)	<0.0001
*HDL, mg/dL (SD)* ^**∗**^	51.5 (15.0)	60.7 (14.2)	44.7 (8.9)	51.9 (11.6)	37.4 (6.0)	40.3 (6.1)	54.2 (10.4)	<0.0001
*Triglycerides, mg/dL* ^**∗**^	108 (156–76)	82 (105–63)	189 (216–171)	106 (126–85)	203 (255–172)	105 (127–83)	185 (221–162)	<0.0001
*Median CAC score with range*	0 (0–6252)	0 (0–6252)	4.08 (0–2791)	3.74 (0–2348)	0 (0–2946)	0 (0–3358)	0 (0–2867)	<0.001
*CAC score > 0(%)* ^**∗**^	2778 (56.50)	1397 (59.9)	82 (47.6)	161 (46.6)	412 (51.9)	516 (56.8)	210 (56.6)	<0.001
*Number of calcified vessels*								<0.0001
*0 (%)* ^**∗**^	2778 (56.4)	1397 (59.9)	82 (47.6)	161 (46.6)	412 (51.9)	516 (56.8)	210 (56.6)	
*1 (%)* ^**∗**^	889 (18.0)	401 (17.2)	36 (20.9)	79 (22.9)	139 (17.5)	158 (17.4)	76 (20.4)	
*2 (%)* ^**∗**^	622 (12.6)	264 (11.3)	18 (10.4)	54 (15.6)	118 (14.8)	131 (14.4)	37 (9.9)	
*3 (%)* ^**∗**^	480 (9.7)	204 (8.7)	27 (15.7)	42 (12.1)	89 (32.8)	82 (9.0)	36 (9.7)	
*4 (%)* ^**∗**^	148 (3.0)	63 (2.7)	9 (5.2)	9 (2.6)	35 (4.4)	20 (2.2)	12 (3.2)	
*Multivessel CAC (%)* ^**∗**^	1250 (25.4)	531 (22.8)	54 (31.4)	105 (30.4)	242 (30.5)	233 (25.6)	85 (22.9)	<0.0001

^*∗*^ *p* < 0.05 for categorical variables using Chi-Squared test or for continuous variables using ANOVA. Percentages may not add up to 100% due to rounding. Continuous variables reported using mean (standard deviation) or median (interquartile range). Normal = normolipidemia, Combined = combined hyperlipidemia, HC = hypercholesterolemia, MetS = metabolic syndrome dyslipidemia, and HTG = hypertriglyceridemia.

**Table 3:** Unadjusted prevalence ratio of multivessel CAC as a function of lipid groups.

Lipid group	PR (95% CI)
Normolipidemia	Ref group
Combined hyperlipidemia^*∗*^	1.37 (1.04–1.82)
Hypercholesterolemia^*∗*^	1.33 (1.08–1.64)
Metabolic syndrome dyslipidemia^*∗*^	1.33 (1.14–1.55)
Low HDL-c	1.12 (0.96–1.31)
Hypertriglyceridemia	1.00 (0.79–1.26)

^*∗*^Statistically significant at *p* < 0.05.

**Table 4:** Adjusted prevalence ratio of multivessel CAC as a function of lipid groups.

	Adjusted PR (95% CI)
*Lipid group*	
Normolipidemia	Ref group
Combined hyperlipidemia^*∗*^	1.41 (1.06–1.87)
Hypercholesterolemia^*∗*^	1.55 (1.26–1.92)
Metabolic syndrome dyslipidemia^*∗*^	1.28 (1.09–1.51)
Low HD-c^*∗*^	1.20 (1.02–1.40)
Hypertriglyceridemia	1.05 (0.83–1.33)
*Age (years)* ^*∗*^	1.05 (1.05–1.06)
*Male sex* ^*∗*^	1.71 (1.49–1.96)
*Race*	
White	Ref group
Asian^*∗*^	1.23 (1.04–1.45)
Black	1.11 (0.88–1.39)
Hispanic	0.89 (0.74–1.07)
*High school education*	1.05 (0.89–1.23)
*Current smoker* ^*∗*^	1.32 (1.12–1.55)
*Hypertension* ^*∗*^	1.19 (1.05–1.34)
*Waist circumference (cm)* ^*∗*^	1.01 (1.0004–1.010757)
*Serum glucose level (mg/dL)*	0.99 (0.99–1.00)
*Serum insulin level (mu/L)*	1.00 (0.99–1.01)
*Serum CRP level (mg/L)*	1.00 (0.99–1.01)
*Serum creatine level (mg/dL)*	0.96 (0.74–1.24)
*Agatston's calcium score* ^*∗*^	1.00 (1.0004–1.0005)

Final model adjusting for age, gender, race, high school education, smoking, hypertension, waist circumference, serum glucose level, serum insulin, serum CRP level, and Agatston's calcium score. All variables are adjusted simultaneously. ^*∗*^Significant at *p* < 0.01.

## Abbreviations

AUC: Areas under receiver operating curve
CAC: Coronary artery calcium
CAD: Coronary artery disease
CCS: Coronary calcium score
CIMT: Carotid intima media thickness
CRP: C-reactive protein
CVD: Cardiovascular disease
(EBT): Electron-beam computed tomography
FRS: Framingham Risk Score
HDL-c: High-density lipoprotein cholesterol
Lp(a): Lipoprotein(a)
LDL-c: Low density lipoprotein cholesterol
MDCT: Multidetector row helical computed tomography
MetS: Dyslipidemia compatible with metabolic syndrome
MESA: Multiethnic Study of Atherosclerosis
TG: Triglycerides.
